# Intact cord resuscitation in newborns with congenital diaphragmatic hernia: insights from a lamb model

**DOI:** 10.3389/fped.2023.1236556

**Published:** 2023-09-06

**Authors:** Baptiste Teillet, Florian Manœuvrier, Céline Rougraff, Capucine Besengez, Laure Bernard, Anne Wojtanowski, Louise Ghesquieres, Laurent Storme, Sébastien Mur, Dyuti Sharma, Kévin Le Duc

**Affiliations:** ^1^ Department of Neonatology, Pôle Femme-Mère-Nouveau-Né, Hôpital Jeanne de Flandre, Centre Hospitalier Universitaire de Lille, Lille, France; ^2^ ULR2694-METRICS: Évaluation des Technologies de Santé et des Pratiques Médicales, Axe Environnement Périnatal et Santé, Centre Hospitalier Universitaire de Lille, Lille, France; ^3^ Department of Pediatry, Centre Hospitalier Universitaire d’Amiens, Lille, France; ^4^ Department of Pediatric Surgery, Jeanne de Flandre Hospital, Centre Hospitalier Universitaire de Lille, Lille, France; ^5^ INSERM CIC-IT 1403, Maison Régionale de la Recherche Clinique, CHRU de, Lille, France; ^6^ Department of Obstetrics, Jeanne de Flandre Hospital, Centre Hospitalier Universitaire de Lille, Lille, France; ^7^ Center for Rare Disease Congenital Diaphragmatic Hernia with the Support of Rare Disease Foundation (Fondation Maladies Rares), Jeanne de Flandre Hospital, Centre Hospitalier Universitaire de Lille, Lille, France

**Keywords:** pulmonary hypoplasia, congenital diaphragmatic hernia (CDH), lamb model, pulmonary hypertension, respiratory—mechanics

## Abstract

**Introduction:**

Congenital diaphragmatic hernia (CDH) is a rare condition characterized by pulmonary hypoplasia, vascular dystrophy, and pulmonary hypertension at birth. Validation of the lamb model as an accurate representation of human CDH is essential to translating research findings into clinical practice and understanding disease mechanisms. This article emphasizes the importance of validating the lamb model to study CDH pathogenesis and develop innovative therapeutics.

**Material and methods:**

At 78 days of gestation, the fetal lamb's left forelimb was exposed through a midline laparotomy and hysterotomy, and a supra diaphragmatic thoracotomy was performed to allow the digestive organs to ascend into the thoracic cavity. At 138 ± 3 days of gestation, lambs were delivered via a cesarean section; then, with umbilical cord intact during 1 hour, the lambs were mechanically ventilated with gentle ventilation in a pressure-controlled mode for 2 h.

**Results:**

CDH lambs exhibited a lower left lung-to-body weight ratio of 5.3 (2.03), *p* < 0.05, and right lung-to-body weight ratio of 8.2 (3.1), *p* < 0.05. They reached lower Vt/kg (tidal volume per kg) during the course of the resuscitation period with 1.2 (0.7) ml/kg at 10 min and 3 (1.65) ml/kg at 60 min (*p* < 0.05). Compliance of the respiratory system was lower in CDH lambs with 0.5 (0.3) ml/cmH_2_O at 60 min (*p* < 0.05) and 0.9 (0.26) ml/cmH_2_O at 120 min (*p* < 0.05). Differences between pre- and postductal SpO_2_ were higher with 15.1% (21.4%) at 20 min and 6.7% (14.5%) at 80 min (*p* < 0.05). CDH lambs had lower differences between inspired and expired oxygen fractions with 4.55% (6.84%) at 20 min and 6.72% (8.57%) at 60 min (*p* < 0.05). CDH lamb had lower left ventricle [2.73 (0.5) g/kg, *p* < 0.05] and lower right ventricle [0.69 (0.8), *p* < 0.05] to left ventricle ratio.

**Discussion:**

CDH lambs had significantly lower tidal volume than control lambs due to lower compliance of the respiratory system and higher airway resistance. These respiratory changes are characteristic of CDH infants and are associated with higher mortality rates. CDH lambs also exhibited pulmonary hypertension, pulmonary hypoplasia, and left ventricle hypoplasia, consistent with observations in human newborns. To conclude, our lamb model successfully provides a reliable representation of CDH and can be used to study its pathophysiology and potential interventions.

## 1. Introduction

Congenital diaphragmatic hernia (CDH), a rare condition that affects one in 3,000 live births, is characterized by a diaphragmatic defect that allows digestive organs to herniate into the thoracic cavity; this results in abnormal lung development, which leads to pulmonary hypoplasia and pulmonary hypertension (PH) (1, 2). On autopsy, a decrease in pre-acinar airway branches is seen in both lungs, and there is a severe reduction in the number of alveoli (3). Despite advances in medical and surgical management, mortality and morbidity rates remain high, at approximately 30% worldwide (4–7). Patients with CDH present with severe respiratory insufficiency and PH at birth, and immediate cord clamping can cause decreased cardiac output and subsequent organ injury (3, 8). Standard neonatal CDH care is based on European consortium guidelines, with immediate intubation before the infant's first breath to avoid digestive distension and optimize ventilation (9). Given the need to improve our understanding of CDH pathogenesis and develop new treatment strategies, animal models play a crucial role in elucidating the underlying mechanisms and allowing us to explore potential interventions.

Lambs, considered the non-primate animal model closest to human physiology, are widely used in studies of congenital diseases and perinatal environments (10–13). Among the animal models used in CDH research, the lamb has emerged as a particularly valuable tool due to its anatomical and physiological similarities to humans (14). Similar to humans, lambs have a muscular diaphragm, allowing examination of diaphragmatic defects and their associated complications. Similar lung development and functions between lambs and humans also make the former an ideal model for studying the respiratory implications of CDH (15). However, despite its widespread use, the need to validate our lamb model to accurately represent human CDH remains paramount. Validating our lamb model is important for several reasons. First, it will ensure that knowledge generated through model studies can be confidently translated into human clinical settings. A robustly validated animal model allows researchers to explore novel therapeutic interventions, assess their safety and efficacy, and optimize treatment strategies for neonatal CDH. Second, validation will facilitate a better understanding of the mechanisms underlying the development of CDH, leading to improved diagnostic and prognostic indicators.

Herein, we tried to emphasize the importance of a clinically relevant lamb model of human CDH because doing so will enhance our understanding of CDH pathogenesis and advance therapeutic approaches, ultimately improving outcomes among infants born with the disease. As the scientific community continues to explore the intricacies of CDH, validating the lamb model has emerged as a crucial step toward a better future for affected patients and their families.

Thus, our objective herein was to better explain pulmonary physiology during the birth in newborns with CDH and pulmonary hypoplasia and to validate our lamb model of this condition.

## 2. Materials and methods

### 2.1. Experimental model

All animal procedures and protocols (experimental research protocol no. 2017121218333678) were approved by the French Ministry of Agriculture (Ministère de l’Agriculture, de la pêche et de l’Alimentation) before the study was carried out in the Department of Experimental Research at Lille University (animal experimentation agreement number D59-35010). Pregnant Ile de France breed ewes were housed in individual pens starting a week before and throughout the procedure.

As previously described by our team, at approximately 78 days of gestation (i.e., during the pseudo-glandular phase of pulmonary development), pregnant ewes were administered general anesthesia induced by xylazine (Sédaxylan, CEVA Santé Animale, Bruxelles, Belgique) and maintained with isoflurane (Aerrane, Baxter, Maurepas, France) while breathing room air and oxygen after intubation. The fetal lamb's left forelimb was exposed through a midline laparotomy and hysterotomy (16–18). The fetal lamb was administered intramuscular buprenorphine (Bupaq, Virbac, France) and subcutaneous lidocaine (Xylocaïne Astrazeneca, Reuil-Malmaison) analgesia, and a supra diaphragmatic thoracotomy was performed to allow the digestive organs to ascend into the thoracic cavity. Afterward, the thoracic cavity was closed, and the amniotic fluid was replaced with physiological serum and amoxicillin before closing the ewe's abdomen. The ewe was kept under surveillance and given analgesia for 24 h after emergence from anesthesia. The ewe remained in the laboratory under daily animal technician supervision for about 2 months until the date for the intact cord fetal resuscitation protocol (19, 20).

### 2.2. Surgical procedure

At 138 ± 3 days of gestation, aseptic procedures were conducted under general anesthesia induced by xylazine (Sédaxylan, CEVA Santé Animale) and maintained with isoflurane (Aerrane, Baxter, Maurepas, France) in a mixture of room air and oxygen after intubation. The fetal lamb's left lower limb was exteriorized through a midline laparotomy and hysterotomy of the pregnant ewe. Prior to the insertion of vascular polyvinyl catheters (4FR, Vygon Ecouen, France) into the aorta via femoral dissection, fetal analgesia was performed with nalbuphine (Nalbuphine, 10 mg IM), and fetal local anesthesia was performed with lidocaine (Lidocaine, 50 mg SC). These procedures aimed to measure aortic pressure at the bifurcation of the common umbilical artery of the abdominal aorta. The left femoral vein catheter was inserted 20 cm into the right atrium.

Catheter patency was maintained by a bolus of heparinized saline, 10 UI/ml (Heparin CHOAY, 5,000 UI, Sanofi-Aventis, Paris, France). At the end of the experimental procedure, animals were euthanized using T61 (Tanax, Intervet Beaucouzé, France) at 3 ml/10 kg body weight for the ewe and 0.3 ml/kg for the lamb.

### 2.3. Delivery and ventilation

A heat lamp was positioned above the table to limit heat loss and lamb cooling. The lamb was dried and placed on warm clothes on a table above the ewe's hooves. Special care was taken to protect the cord from drying and to prevent its stretching, kinking, or compression.The sedated pregnant ewe was not administered an oxytocin injection throughout the resuscitation phase to prevent placental delivery. The ewe was sedated with isoflurane, and the neonatal lamb was resuscitated with the cord intact. Thus, the lamb was anesthetized and sedated and did not breathe spontaneously.

**Figure 1:**
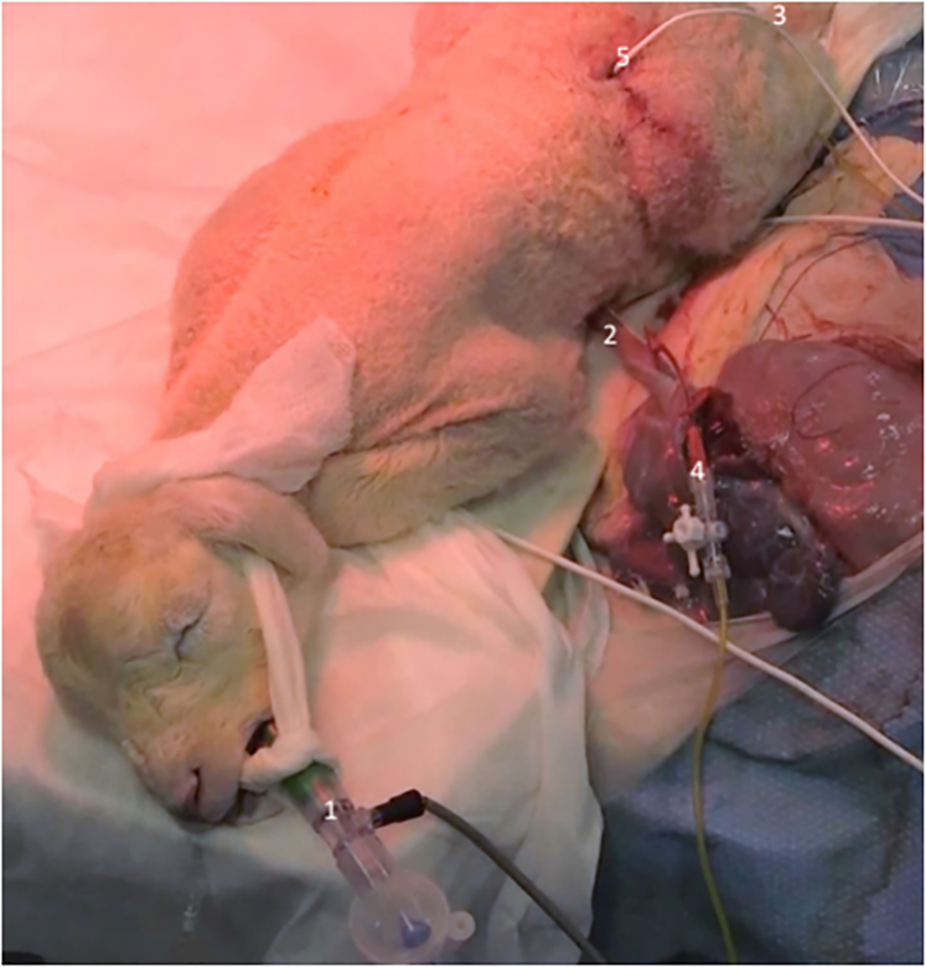
Intact cord resuscitation of a newborn lamb with tracheal intubation (1), umbilical cord (2), femoral catheter (3), umbilical veinous catheter (4), and ultrasonic flow probe (5).

As shown in Figure 1, after pharyngeal suctioning, the lamb was intubated with a 4.5-mm cuffed endotracheal tube (Rüschelit, Teleflex medical, Wayne, PA, USA). First, 30 s of sustained inflation at 30 cmH_2_O was performed, after which the lamb was mechanically administered gentle ventilation (Infant Star 950, Covidien, Dublin, Ireland) with a monitor (SLE 2100, Malmesbury, Wiltshire, UK) in a pressure-controlled mode [positive end-expiratory pressure: 5 cmH_2_O, peak inspiratory pressure (PIP): 24 cmH_2_O, FR: 60/min, FiO_2_: 1 = 100%] for 2 h. After shaving the right and left frontpaw, preductal and postductal SpO_2_ sensors were used to continuously record blood oxygen saturation. Mechanical ventilation was adjusted to the target 40–60 mmHg PCO_2_. If PCO_2_ was >60 mmHg, inspiratory pressure was increased by 5 cmH_2_O. If PCO_2_ was >80 mmHg, inspiratory pressure was increased by 5 cmH_2_O, and the respiratory rate was increased by 20 breaths per minute, only if the increase of respiratory rate allowed sufficient expiratory time on respiratory flow curves to avoid intrinsic PEEP and achieving appropriate tidal volume (TV). PEEP was not adjusted in our experimental protocol to avoid overdistension, as it has been shown that a lower level of PEEP results in better oxygenation (21). The SpO_2_ target was between 92% and 99%. FiO_2_ was adjusted every 5 min to achieve the target. We did not use any curare muscle relaxant during our experimentation. Rectal temperature was recorded continuously throughout the resuscitation.

### 2.4. Respiratory assessment

Arterial blood samples were analyzed at 20 min intervals throughout the resuscitation process. Key respiratory parameters, including TV(per kg), airway pressures [positive end-expiratory pressure (PEEP); PIP], and lung compliance and resistance, were recorded at the beginning of ventilation and then every 10 min during the 2-h resuscitation period. Peripheral oxygen saturation levels were also recorded before and after ductus arteriosus. A ventilator (Infant Star 950, Covidien, Dublin, Ireland) with a monitor (SLE 2100) was used to estimate compliance and resistance of the respiratory system through the collection of pressure and airflow data. Herein, compliance (*C*) is the change in lung volume (Δ*V*) per unit change in transpulmonary pressure (Δ*P*) (compliance formula: *C* = Δ*V*/Δ*P*). Respiratory system resistance (*R*) was determined as pressure difference across the airways (Δ*P*) divided by the flow rate (*Q*) (resistance formula: *R* = Δ*P*/*Q*). Digital filtering techniques were used to calculate variations in the data. The least-square estimation algorithm was then used to estimate lung compliance and resistance in real time.

### 2.5. Postmortem examination

Confirmation of the presence of a defect in the diaphragm and herniation of visceral organs was made through postmortem examination. The lungs were weighed, and the results were expressed as a ratio to the body weight (i.e., wet lung-to-body weight ratio). We examined the wet-to-dry lung ratio to estimate the presence of lung edema after the 2-h resuscitation period. The weights of the total heart and left and right ventricles were measured. Both CDH and control lamb hearts were dissected systematically by the same individual. The left and right ventricles were dissected and weighed in a standard manner as previously described (22).

### 2.6. Statistical analysis

Variables were collected immediately before and after starting mechanical ventilation. All statistical analyses were conducted using SPSS version 24 (IBM Corporation, Armonk, NY, USA). Continuous variables are reported as mean ± standard deviation after checking for distribution normality with the Shapiro–Wilk test. The nonparametric Friedman and Wilcoxon distribution-free tests were used to assess the significance of differences in respiratory and hemodynamic measures.

## 3. Results

### 3.1. Surgery

Sixteen pregnant ewes underwent surgery at approximately 78 days of gestation. Intrauterine fetal death before ventilation or preterm delivery occurred in three cases (18%). Among 16 fetuses with surgical creation of CDH, five (31%) had healed CDH and were included in the control lamb group. Five fetuses (31%) did not undergo the entire resuscitation process due to death or the inability to record appropriate data. Therefore, six lambs with CDH (37%) were subjected to a 2-h resuscitation period. Among the 19 control lambs, nine (47%) were subjected to a 2-h resuscitation period.

### 3.2. Respiratory function

**Figure 2:**
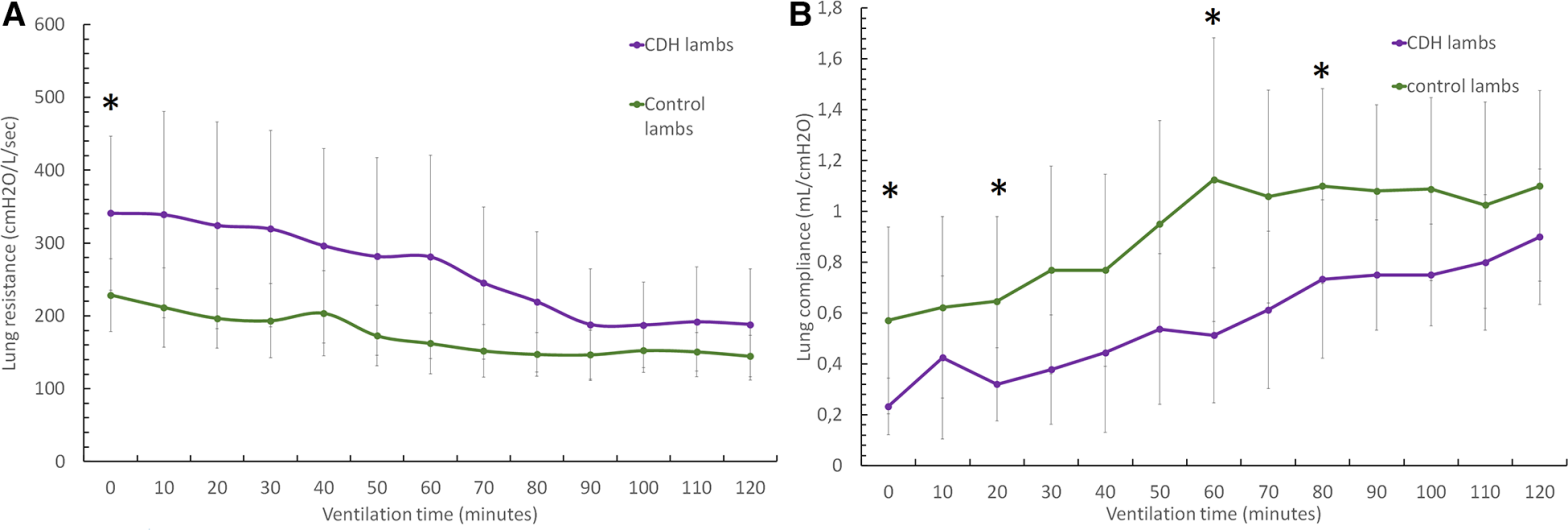
Evolution of respiratory parameters during the 2-h resuscitation. (**A**) Lung resistance evolution over time and (**B**) lung compliance evolution over time. Groups shown are CDH lambs and control lambs. The data are presented as mean ± SEM and significantly accepted when *p* < 0.05 tested at four time points during resuscitation (at 0, 20, 60, and 80 min). Asterisk (*) indicates statistically significant differences between CDH and control lambs at individual time points.

The CDH lambs had higher respiratory system resistance during the first minutes of resuscitation [341 (105) vs. 228 (49) cmH_2_O/L/s at 50 min (*p* < 0.05)]. As shown in Figure 2, lung compliance increased in both groups, although much less so in CDH lambs, and was significantly reduced after 2 h of resuscitation. We observed respiratory system compliance of 0.32 ± 0.14 ml/cmH_2_O in CDH lambs and 0.64 ± 0.33 ml/cmH_2_O in control lambs at 20 min (*p* < 0.05), 0.51 ± 0.26 vs. 1.12 (0.6) ml/cmH_2_O at 60 min (*p* < 0.05), 0.73 ± 0.2 vs. 1.1 ± 0.38 ml/cmH_2_O at 80 min (*p* = 0.5), and 0.9 ± 0.26 vs. 1.1 ± 0.375 ml/cmH_2_O at 120 min (*p* < 0.05).

**Figure 3:**
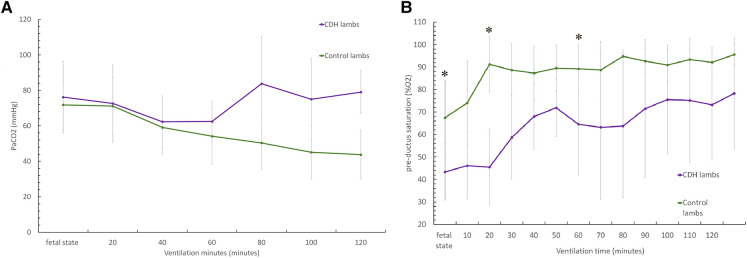
Evolution of respiratory parameters during the 2-h resuscitation. (**A**) PaCO_2_ level's evolution over time and (**B**) preductus arteriosus saturation level's evolution over time. Groups shown are CDH lambs and control lambs. The data are presented as mean ± SEM and significantly accepted when *p < *0.05 tested at four time points during resuscitation (at 0, 20, 60, and 80 min). Asterisk (*) indicates statistically significant differences between CDH and control lambs at individual time points.

**Figure 4:**
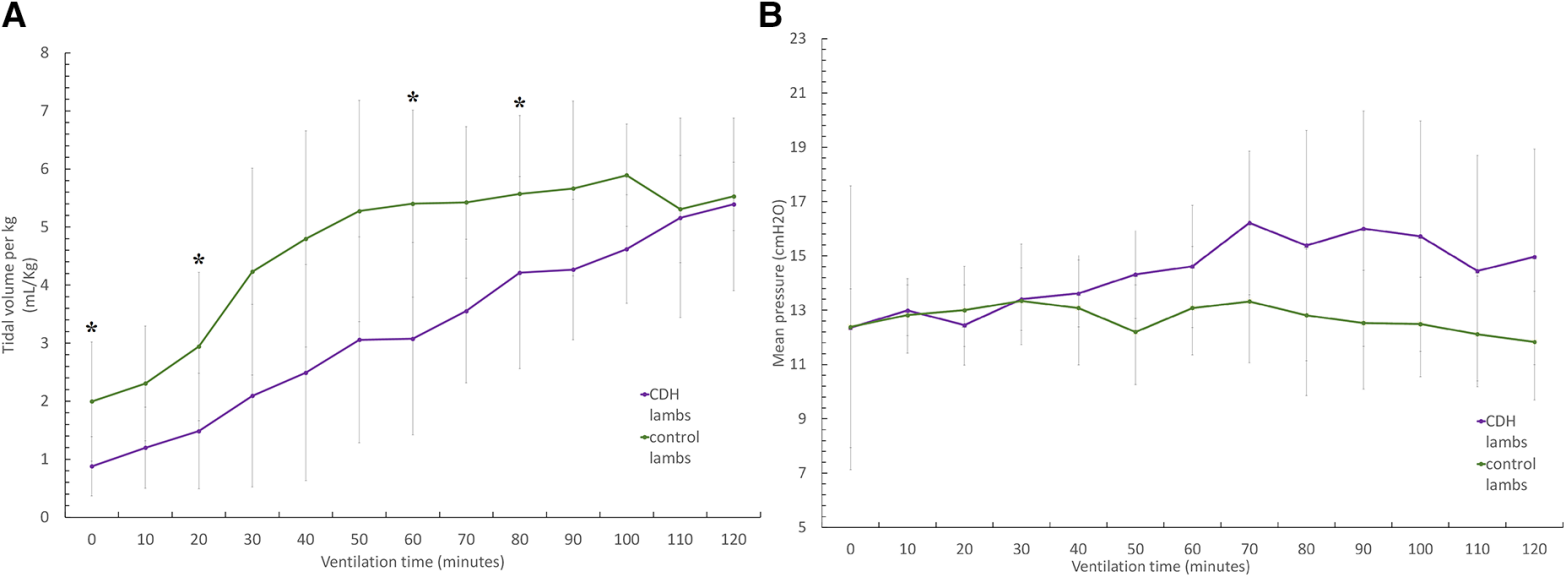
Evolution of ventilatory parameters during the 2-h resuscitation. (**A**) Evolution of tidal volume to lamb's weight (TV/kg) over time and (**B**) evolution of mean ventilatory pressure over time. Groups shown are CDH lambs and control lambs. The data are presented as mean ± SEM and significantly accepted when *p* < 0.05 tested at four time points during resuscitation (at 0, 20, 60, and 80 min). Asterisk (*) indicates statistically significant differences between CDH and control lambs at individual time points.

**Figure 5:**
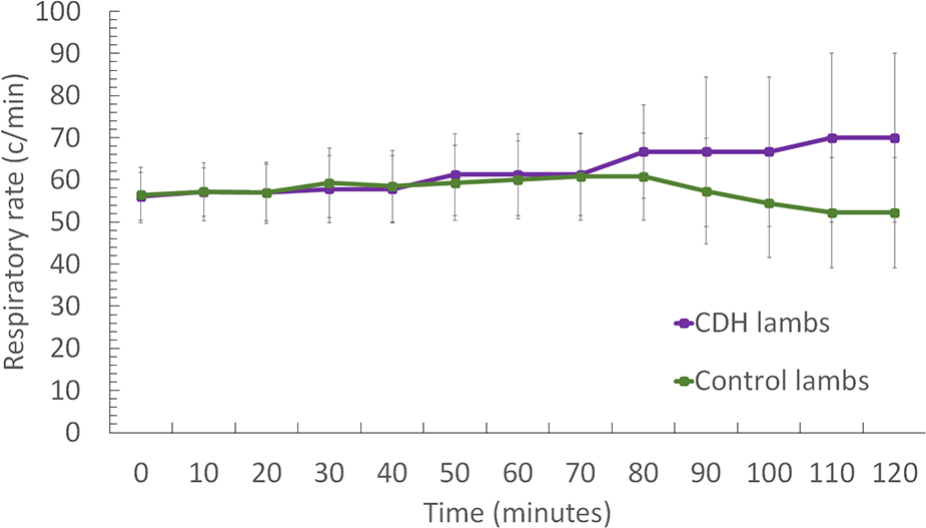
Evolution of respiratory rate during the 2-h resuscitation. Groups shown are CDH lambs and control lambs. The data are presented as median ± SEM and significance accepted when *p* < 0.05 tested at four time points during resuscitation (0, 20, 60, and 80 min). Asterisk (*) indicated significant differences between CDH and control lambs at individual time points.

CDH lambs also exhibit significantly lower preductal saturation, with 43 ± 12.5% vs. 67.5 ± 16.6% at baseline (*p* < 0.05), 58 ± 18.6% vs. 88.6 ± 12% at 20 min, and 63.2 ± 32% vs. 88.7 ± 12.8% at 60 min (Figure 3). As shown in Figure 4, CDH lambs reached significantly lower Vt/kg throughout resuscitation, with 0.87 ± 0.5 vs. 2 ± 1.02 ml/kg at baseline, 1.4 ± 0.9 vs. 2.9 ± 1.2 ml/kg at 20 min, and 3.1 ± 1.8 vs. 5.4 ± 1.6 ml/kg at 60 min (all *p*’s < 0.05). As can be seen in Figure 5, there were no significant differences in the respiratory rate during our experiment. Unlike control lambs, there was no significant improvement in TV before 30 min of resuscitation in CDH lambs. At the end of the 2 h of ventilation, CDH lambs reached a similar Vt/kg at the price of greater mean ventilation pressure (14.9 ± 3.9 vs. 11.89 ± 1.9 cmH_2_O; *p* < 0.05). CDH lambs showed a lower pH level at the end of resuscitation [7.12 (0.1) vs. 7.28 (0.15); *p* < 0.05].

**Figure 6:**
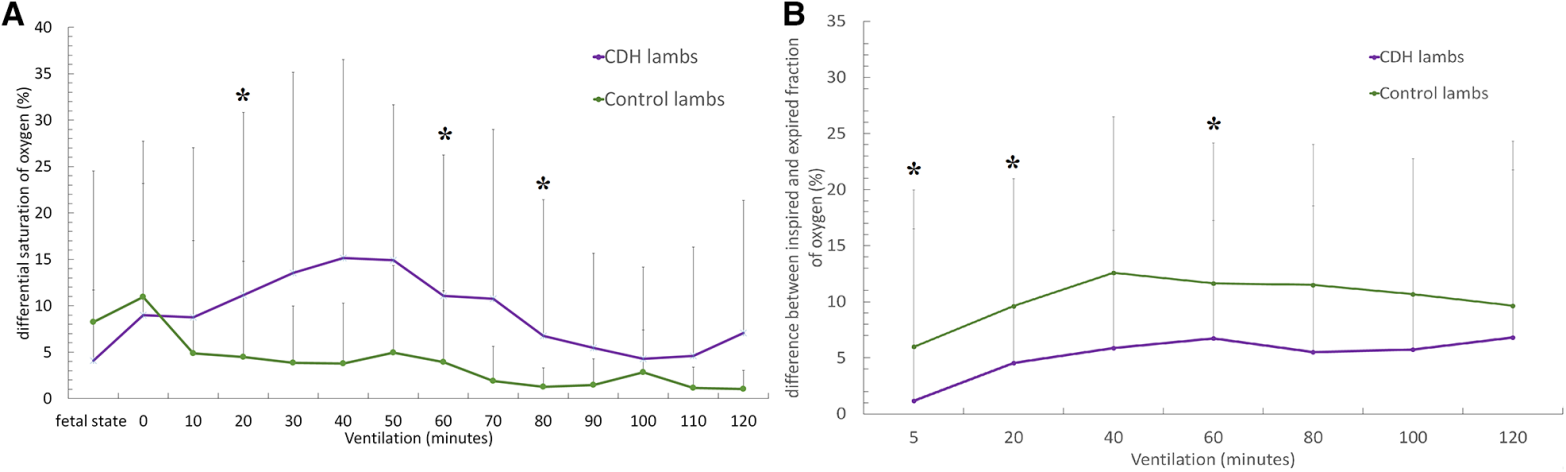
(**A**) Evolution of the difference between preductus and postductus arteriosus oxygen saturation levels during the two hours resuscitation. (**B**) Evolution of the difference between inspired and expired fractions of oxygen. Groups shown are CDH lambs and control lambs. The data are presented as mean ± SEM and significantly accepted when *p* < 0.05 tested at four time points during resuscitation (at 0, 20, 60, and 80 min). Asterisk (*) indicates statistically significant differences between CDH and control lambs at individual time points.

As shown in Figure 6, CDH lambs exhibited a higher saturation differential at 20 (15.1 ± 21.4% vs. 3.78 ± 6.48%), 60 (11.07 ± 15.1% vs. 3.94 ± 7.68%), and 80 min of resuscitation (6.7 ± 14.5% vs. 1.2 ± 2.05%) (all, *p*’s < 0.05). CDH lambs exhibited significantly less difference between inspiratory and expiratory oxygen fractions, 1.1 ± 4.2% vs. 5.97 ± 21.8% at 5 min; 4.55 ± 6.84% vs. 12.58 ± 9.76% at 20 min, and 6.72 ± 8.57% vs. 11.6 ± 8.98% at 60 min (all *p*’s < 0.05).

### 3.3. Postmortem examination

Fifteen lambs were confirmed to have a diaphragmatic defect that included the stomach, small intestine, and, in some cases, the liver and spleen. It is crucial to emphasize that our analysis only included animals with clearly documented herniation in the left chest during an autopsy.

**Figure 7:**
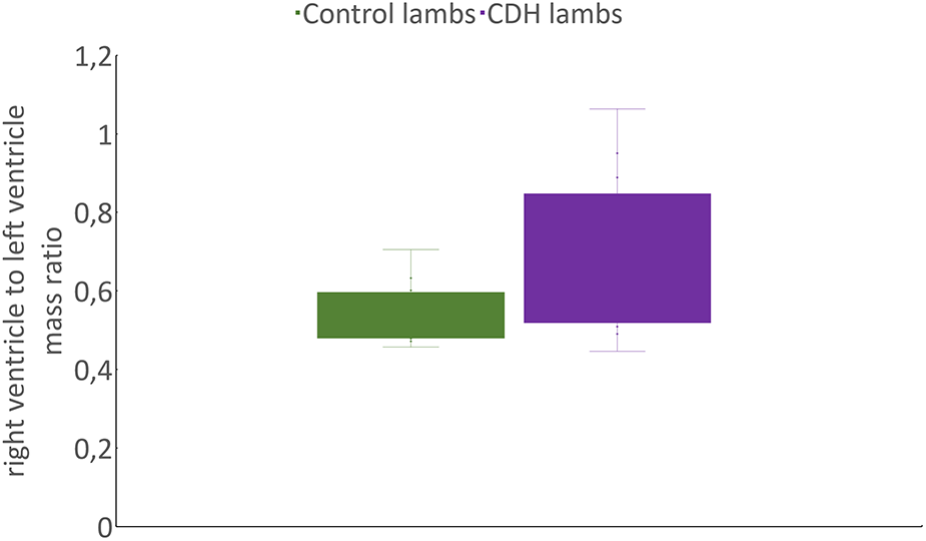
Comparison of RV/LV mass based on lamb's weight ratio between CDH and control lambs. RV, right ventricle; LV, left ventricle.

Compared with the control lambs, CDH lambs exhibited a significant reduction in the left wet lung-to-body weight ratio (5.3 ± 2.03 vs. 13.7 ± 2 g/kg; *p* < 0.05) and right wet lung-to-body weight ratio (8.2 ± 3.1 vs. 19.8 ± 3.1 g/kg; *p* < 0.05) (Table 1). In addition, as shown in Figure 7, the weight of the left heart of CDH lambs, based on lamb’ weight, was significantly reduced (2.5 ± 0.5 vs. 3.5 ± 0.3 g/kg; *p* < 0.05) as was the right ventricle to left ventricle mass ratio (0.69 ± 0.8 vs. 0.52 ± 0.06 g/kg; *p* < 0.05).

**Table 1:** Necropsy's examination record (values represent medians and interquartiles).

	Control lambs (*n* = 9)	CDH lambs (*n* = 6)	*p* (< 0.05)
Left ventricle to lamb’s weight (g/kg)	3.5 (0.3)	2.57 (0.5)	**0** **.** **04**
Right ventricle to lamb’s weight (g/kg)	1.85 (0.2)	1.76 (0.4)	0.53
Heart mass to lamb’s weight (g/kg)	12.1 (9.7)	6.4 (1.05)	0.53
Left lung mass to lamb’s weight (g/kg)	13.7 (2)	5.3 (2.03)	**0**.**003**
Right lung mass to lamb’s weight (g/kg)	19.8 (3.1)	8.2 (3.1)	**0**.**005**

Bold values indicate significant data, *p* < 0.05.

## 4. Discussion

In this study, we successfully validated our lamb CDH model for studying neonatal adaptation to extrauterine life. CDH lambs exhibited significant differences in TV during most of the resuscitation period due to lower lung compliance and higher airway resistance, which reached the levels of control lambs at the end of resuscitation. This is consistent with previous observations that functional residual capacity and Vt recruitment are significantly slower in CDH lungs and that gas exchange improved more slowly in CDH lambs (23, 24). This lower respiratory system compliance, which is typical of infants with CDH, is associated with higher morbidity and mortality (25). To prevent any potential bias in our study, we intentionally avoided using a curare muscle relaxant during the experimentation. This decision was based on previous research indicating that curare administration can lead to compliance changes and increased mortality in infants with CDH (26, 27).

Lung changes in CDH include decreased terminal branching of the bronchioles, leading to acinar hypoplasia with fewer alveoli, reduced gas exchange area, and increased interstitial tissue (28, 29). We can hypothesize that alveolar recruitment would eventually have matched ventilation and perfusion after prolonged ventilation.

In addition, our animal model exhibits significant loss, and there are two potential explanations for this. First, the severity of the lung hypoplasia in some CDH lambs resulted in maladaptation to extrauterine life and severe hypoxia. Second, this model has inherent mortality, with a reported perioperative mortality rate between 30% and 50% in the literature (15). Third, placements of catheters through the umbilical cord and ultrasound probe around the umbilical artery have been associated with death or failure to obtain accurate data throughout the experiment. Nevertheless, these findings are consistent with previous reports regarding the difficulty of this model and mimic the severity of the disease (15, 30).

Arterial blood gas status during the transition to extrauterine life is a crucial prognostic factor of long-term survival. Herein, we observed that CDH lambs required high levels of oxygen (FiO_2_) and carbon dioxide (PaCO_2_). CDH lambs also demonstrated higher levels of differential oxygen saturation, suggesting higher pulmonary vascular resistance (PVR) and a greater right-to-left shunt through the ductus arteriosus. This differential was initially lower in CDH lambs because of lower preductus arteriosus oxygen saturation during the initial minutes of resuscitation. The disparity in the fraction of oxygen extracted from the lungs of CDH and control lambs cannot be entirely attributed to the reduced oxygen consumption of the former. This suggests reduced perfusion of the pulmonary vessels caused by high PVR, which is essential for efficient gas exchange. We also observed lower pH in lambs with CDH, which is known to increase PVR and worsen adaptation to extrauterine life (31–33). In CDH, a reduced pulmonary vascular cross-sectional area combined with extensive hypermuscularization and neomuscularization of distal pulmonary vessels leads to higher resistance within peripheral pulmonary vessels, resulting in persistent PH (PPHN) of the newborn (8, 22, 34). In addition, altered vasoreactivity may contribute to a reversible component of PPHN due to an imbalance of autonomic innervation, impairment of endothelium-dependent relaxation, and an imbalance between vasoconstrictor and vasodilator mediators (35–37).

Herein, we successfully modeled the pulmonary hypoplasia observed in newborns with CDH (38). This model included both the right and left lungs, providing a comprehensive representation of the disease. We also discovered notable left ventricular hypoplasia evidenced by a significantly lower ratio of right to left ventricle mass in relation to the lamb weight. This finding aligns with previous reports of left ventricular hypoplasia and cardiac dysfunction in human newborns with CDH (39, 40). Moreover, our investigation revealed no differences in the lung wet/dry ratios following the 2-h resuscitation. This underscores the advantages of our gentle ventilation strategy, which is consistent with human neonatal care (9). These results emphasize the translational relevance of our lamb model, which accurately recapitulates key aspects of CDH pathophysiology and demonstrates the potential benefits of our approach to respiratory support (39).

This animal model indeed already exists in the literature; however, it remains necessary to validate our model of diaphragmatic hernia. Moreover, to our knowledge, this is the first study to investigate the pulmonary function of newborns with a diaphragmatic hernia in the context of intact cord resuscitation. The lamb model has been used extensively to study congenital diseases such as CDH. Our results are consistent with several studies showing that lamb models exhibit the main features of the CDH. Kashyap et al. showed that lambs with CDH have small, noncompliant lungs, poor cerebral oxygenation, and respiratory acidosis, reproducing the clinical features of infants with CDH (41). DeKoninck et al. then showed that tracheal occlusion (i.e., the fetoscopic endoluminal tracheal occlusion or FETO procedure) increases lung size and pulmonary blood flow (23). Similarly, Bhatt et al. showed that delaying cord clamping after the beginning of ventilation improves cardiovascular function at birth in preterm lambs (42). These studies have led randomized clinical FETO trials to treat severe CDH in the human fetus (43), highlighting the importance of validating lamb models in the laboratory, using high-quality empirical tests, for facilitating innovative management of patients with CDH.

In our laboratory, animal experiments have led to the implementation of human clinical trials, including the study of the Efficacy of Intact Cord Resuscitation Compared to Immediate Cord Clamping on Cardiorespiratory Adaptation at Birth in Infants with Isolated Congenital Diaphragmatic Hernia (CHIC) (19, 20). However, these experiences have raised numerous questions regarding this resuscitation technique, underscoring the need to refine and validate our model to address them.

Our study has some limitations. Some of our lambs exhibited spontaneous healing during gestation, with no evidence of diaphragmatic hernia found during necropsy as shown in other studies (44). This healing process can be attributed to various factors. First, the fetus has a remarkable healing capacity due to the presence of high concentrations of growth factors, including TGF-beta and interleukin-10. Studies by Longaker et al. have demonstrated that lambs can heal diaphragmatic wounds during gestation (45, 46). Second, it is possible that during the closure of the thoracic wall and the reintegration of the fetus into the womb, there is an application of thoracic pressure, which helps reduce the occurrence of diaphragmatic hernia. Furthermore, the liver was not always elevated in the thoracic area to prevent perioperative deaths due to liver injury or the creation of an extremely severe hernia. Finally, it may be beneficial to remove a portion of the diaphragm rather than just a section to ensure a more accurate and appropriate model.

In conclusion, by validating our lamb model as an effective tool for studying CDH, we contribute to the growing evidence supporting its use in research and therapeutic development. Furthermore, our findings suggest that the lamb model may serve as a valuable resource for investigating the underlying mechanisms of CDH and evaluating potential interventions to improve clinical outcomes in affected infants. Based on these findings, our group will use this model to study the relevance of intact cord resuscitation and its optimal setting to further improve neonatal CDH outcomes.

## Data Availability

The raw data supporting the conclusions of this article will be made available by the authors, without undue reservation.

## References

[1] Lally KP. Congenital diaphragmatic hernia. Curr Opin Pediatr. (2002) 14(4):486–90. 10.1097/00008480-200208000-00022 12130916

[2] Greer JJ. Current concepts on the pathogenesis and etiology of congenital diaphragmatic hernia. Respir Physiol Neurobiol. (2013) 189(2):232–40. 10.1016/j.resp.2013.04.015 23665522

[3] Keller RL. Antenatal and postnatal lung and vascular anatomic and functional studies in congenital diaphragmatic hernia: implications for clinical management. Am J Med Genet C Semin Med Genet. (2007) 145C(2):184–200. 10.1002/ajmg.c.30130 17436304

[4] Flemmer AW Thio M Wallace MJ Lee K Kitchen MJ Kerr L Lung hypoplasia in newborn rabbits with a diaphragmatic hernia affects pulmonary ventilation but not perfusion. Pediatr Res. (2017) 82(3):536–43. 10.1038/pr.2017.91 28399114 PMC5605670

[5] Jani J Nicolaides KH Keller RL Benachi A Peralta CFA Favre R Observed to expected lung area to head circumference ratio in the prediction of survival in fetuses with isolated diaphragmatic hernia. Ultrasound Obstet Gynecol. (2007) 30(1):67–71. 10.1002/uog.4052 17587219

[6] Cannie M Jani JC De Keyzer F Devlieger R Van Schoubroeck D Witters I Fetal body volume: use at MR imaging to quantify relative lung volume in fetuses suspected of having pulmonary hypoplasia. Radiology. (2006) 241(3):847–53. 10.1148/radiol.2413051228 17053198

[7] Cannie M Jani J Meersschaert J Allegaert K Done’ E Marchal G Prenatal prediction of survival in isolated diaphragmatic hernia using observed to expected total fetal lung volume determined by magnetic resonance imaging based on either gestational age or fetal body volume. Ultrasound Obstet Gynecol. (2008) 32(5):633–9. 10.1002/uog.6139 18792417

[8] Gupta VS Harting MT. Congenital diaphragmatic hernia-associated pulmonary hypertension. Semin Perinatol. (2020) 44(1):151167. 10.1053/j.semperi.2019.07.006 31519366

[9] Snoek KG Reiss IKM Greenough A Capolupo I Urlesberger B Wessel L Standardized postnatal management of infants with congenital diaphragmatic hernia in Europe: the CDH EURO consortium consensus—2015 update. Neonatology. (2016) 110(1):66–74. 10.1159/000444210 27077664

[10] Lewis NA Holm BA Swartz D Sokolowski J Rossman J Glick PL. Antenatal vitamin a decreases ventilation-induced lung injury in the lamb model of congenital diaphragmatic hernia. Asian J Surg. (2006) 29(3):193–7. 10.1016/S1015-9584(09)60086-5 16877224

[11] Jelin EB Etemadi M Encinas J Schecter SC Chapin C Wu J Dynamic tracheal occlusion improves lung morphometrics and function in the fetal lamb model of congenital diaphragmatic hernia. J Pediatr Surg. (2011) 46(6):1150–7. 10.1016/j.jpedsurg.2011.03.049 21683214 PMC3128884

[12] Larson AC Didier R Daszewska-Smith G Chang J Sridharan A Agarwal D The fetal lamb model of congenital diaphragmatic hernia shows altered cerebral perfusion using contrast enhanced ultrasound. J Pediatr Surg. (2022) 57(6):991–8. 10.1016/j.jpedsurg.2022.02.006 35346482

[13] Garabedian C Aubry E Sharma D Bleu G Clermont-Hama Y Ghesquière L Exploring fetal response to acidosis in ewes: choosing an adequate experimental model. J Gynecol Obstet Hum Reprod. (2018) 47(8):397–403. 10.1016/j.jogoh.2018.04.007 29654942

[14] Hooper SB Te Pas AB Polglase GR Wyckoff M. Animal models in neonatal resuscitation research: what can they teach US? Semin Fetal Neonatal Med. (2018) 23(5):300–5. 10.1016/j.siny.2018.07.002 30001819

[15] Wilcox DT Irish MS Holm BA Glick PL. Animal models in congenital diaphragmatic hernia. Clin Perinatol. (1996) 23(4):813–22. 10.1016/S0095-5108(18)30211-2 8982573

[16] Sharma D Aubry E Ouk T Houeijeh A Houfflin-Debarge V Besson R Effects of eicosapentaenoic acid (EPA) and docosahexaenoic acid (DHA) on fetal pulmonary circulation: an experimental study in fetal lambs. Nutrients. (2017) 9(7):E761. 10.3390/nu9070761 PMC5537875 28714905

[17] Houeijeh A Aubry E Coridon H Montaigne K Sfeir R Deruelle P Effects of n-3 polyunsaturated fatty acids in the fetal pulmonary circulation. Crit Care Med. (2011) 39(6):1431–8. 10.1097/CCM.0b013e31821204fb 21378553

[18] Aubry E Fayoux P Jani J Deprest J Deruelle P Houfflin-Debarge V Tracheal occlusion alters pulmonary circulation in the fetal lamb with normally developing lungs. J Pediatr Surg. (2013) 48(3):481–7. 10.1016/j.jpedsurg.2012.08.024 23480900

[19] Le Duc K Mur S Rakza T Boukhris MR Rousset C Vaast P Efficacy of intact cord resuscitation compared to immediate cord clamping on cardiorespiratory adaptation at birth in infants with isolated congenital diaphragmatic hernia (CHIC). Child Basel Switz. (2021) 8(5):339. 10.3390/children8050339 PMC8146982 33925985

[20] Le Duc K Aubry E Mur S Besengez C Garabedian C De Jonckheere J Changes in umbilico-placental circulation during prolonged intact cord resuscitation in a lamb model. Child Basel Switz. (2021) 8(5):337. 10.3390/children8050337 PMC8145708 33925880

[21] Guevorkian D Mur S Cavatorta E Pognon L Rakza T Storme L. Lower distending pressure improves respiratory mechanics in congenital diaphragmatic hernia complicated by persistent pulmonary hypertension. J Pediatr. (2018) 200:38–43. 10.1016/j.jpeds.2018.04.027 29793868

[22] Karamanoukian HL Glick PL Wilcox DT O’Toole SJ Rossman JE Azizkhan RG. Pathophysiology of congenital diaphragmatic hernia. XI: anatomic and biochemical characterization of the heart in the fetal lamb CDH model. J Pediatr Surg. (1995) 30(7):925–8; discussion 929. 10.1016/0022-3468(95)90314-3 7472946

[23] DeKoninck PLJ Crossley KJ Kashyap AJ Skinner SM Thio M Rodgers KA Effects of tracheal occlusion on the neonatal cardiopulmonary transition in an ovine model of diaphragmatic hernia. Arch Dis Child Fetal Neonatal Ed. (2019) 104(6):F609–16. 10.1136/archdischild-2018-316047 30728180

[24] Bratu I Flageole H Laberge JM Kovacs L Faucher D Piedboeuf B. Lung function in lambs with diaphragmatic hernia after reversible fetal tracheal occlusion. J Pediatr Surg. (2004) 39(10):1524–31. 10.1016/j.jpedsurg.2004.06.024 15486898

[25] Kavvadia V Greenough A Laubscher B Dimitriou G Davenport M Nicolaides KH. Perioperative assessment of respiratory compliance and lung volume in infants with congenital diaphragmatic hernia: prediction of outcome. J Pediatr Surg. (1997) 32(12):1665–9. 10.1016/S0022-3468(97)90502-9 9433995

[26] Murthy V D’Costa W Nicolaides K Davenport M Fox G Milner AD Neuromuscular blockade and lung function during resuscitation of infants with congenital diaphragmatic hernia. Neonatology. (2012) 103(2):112–7. 10.1159/000342332 23182955

[27] Weems MF Grover TR Seabrook R DiGeronimo R Gien J Keene S Analgesia, sedation, and neuromuscular blockade in infants with congenital diaphragmatic hernia. Am J Perinatol. (2023) 40(4):415–23. 10.1055/s-0041-1729877 34044457

[28] George DK Cooney TP Chiu BK Thurlbeck WM. Hypoplasia and immaturity of the terminal lung unit (acinus) in congenital diaphragmatic hernia. Am Rev Respir Dis. (1987) 136(4):947–50. 10.1164/ajrccm/136.4.947 3662245

[29] Pierro M Thébaud B. Understanding and treating pulmonary hypertension in congenital diaphragmatic hernia. Semin Fetal Neonatal Med. (2014) 19(6):357–63. 10.1016/j.siny.2014.09.008 25456753

[30] Jiménez JA Eixarch E DeKoninck P Bennini JR Devlieger R Peralta CF Balloon removal after fetoscopic endoluminal tracheal occlusion for congenital diaphragmatic hernia. Am J Obstet Gynecol. (2017) 217(1):78.e1–11. 10.1016/j.ajog.2017.02.041 28267443

[31] Fuloria M Aschner JL. Persistent pulmonary hypertension of the newborn. Semin Fetal Neonatal Med. (2017) 22(4):220–6. 10.1016/j.siny.2017.03.004 28342684

[32] Walsh-Sukys MC Tyson JE Wright LL Bauer CR Korones SB Stevenson DK Persistent pulmonary hypertension of the newborn in the era before nitric oxide: practice variation and outcomes. Pediatrics. (2000) 105(1 Pt 1):14–20. 10.1542/peds.105.1.14 10617698

[33] Malik AB Mewmark JM. Adrenergic mechanisms and the pulmonary vascular response to respiratory acidosis. Respir Int Rev Thorac Dis. (1976) 33(3):179–87. 10.1159/000193732 935680

[34] O’Toole SJ Irish MS Holm BA Glick PL. Pulmonary vascular abnormalities in congenital diaphragmatic hernia. Clin Perinatol. (1996) 23(4):781–94. 10.1016/S0095-5108(18)30209-4 8982571

[35] Lath NR Galambos C Rocha AB Malek M Gittes GK Potoka DA. Defective pulmonary innervation and autonomic imbalance in congenital diaphragmatic hernia. Am J Physiol Lung Cell Mol Physiol. (2012) 302(4):L390–398. 10.1152/ajplung.00275.2011 22114150 PMC3774131

[36] Schmidt AF Rojas-Moscoso JA Gonçalves FLL Gallindo RM Mónica FZ Antunes E Increased contractility and impaired relaxation of the left pulmonary artery in a rabbit model of congenital diaphragmatic hernia. Pediatr Surg Int. (2013) 29(5):489–94. 10.1007/s00383-012-3238-8 23269641

[37] Shinkai T Shima H Solari V Puri P. Expression of vasoactive mediators during mechanical ventilation in nitrofen-induced diaphragmatic hernia in rats. Pediatr Surg Int. (2005) 21(3):143–7. 10.1007/s00383-004-1310-8 15756563

[38] Chinoy MR. Pulmonary hypoplasia and congenital diaphragmatic hernia: advances in the pathogenetics and regulation of lung development. J Surg Res. (2002) 106(1):209–23. 10.1006/jsre.2002.6390 12127828

[39] Patel N Massolo AC Kipfmueller F. Congenital diaphragmatic hernia-associated cardiac dysfunction. Semin Perinatol. (2020) 44(1):151168. 10.1053/j.semperi.2019.07.007 31420110

[40] Siebert JR Haas JE Beckwith JB. Left ventricular hypoplasia in congenital diaphragmatic hernia. J Pediatr Surg. (1984) 19(5):567–71. 10.1016/S0022-3468(84)80105-0 6502429

[41] Kashyap AJ Crossley KJ DeKoninck PLJ Rodgers KA Thio M Skinner SM Neonatal cardiopulmonary transition in an ovine model of congenital diaphragmatic hernia. Arch Dis Child Fetal Neonatal Ed. (2019) 104(6):F617–23. 10.1136/archdischild-2018-316045 30728182

[42] Bhatt S Alison BJ Wallace EM Crossley KJ Gill AW Kluckow M Delaying cord clamping until ventilation onset improves cardiovascular function at birth in preterm lambs. J Physiol. (2013) 591(8):2113–26. 10.1113/jphysiol.2012.250084 23401615 PMC3634523

[43] Deprest JA Nicolaides KH Benachi A Gratacos E Ryan G Persico N Randomized trial of fetal surgery for severe left diaphragmatic hernia. N Engl J Med. (2021) 385(2):107–18. 10.1056/NEJMoa2027030 34106556 PMC7613453

[44] Basurto D Sananès N Bleeser T Valenzuela I De Leon N Joyeux L Safety and efficacy of smart tracheal occlusion device in diaphragmatic hernia lamb model. Ultrasound Obstet Gynecol. (2021) 57(1):105–12. 10.1002/uog.23135 33012007 PMC7613565

[45] Longaker MT Whitby DJ Jennings RW Duncan BW Ferguson MW Harrison MR Fetal diaphragmatic wounds heal with scar formation. J Surg Res. (1991) 50(4):375–85. 10.1016/0022-4804(91)90206-2 2020189

[46] Larson BJ Longaker MT Lorenz HP. Scarless fetal wound healing: a basic science review. Plast Reconstr Surg. (2010) 126(4):1172–80. 10.1097/PRS.0b013e3181eae781 20885241 PMC4229131

